# Pearls and Pitfalls of Epicardial Echocardiography for Implantation of Impella CP Devices in Ovine Models

**DOI:** 10.1007/s12265-024-10555-1

**Published:** 2024-09-10

**Authors:** Konstantin Yastrebov, Laurencie M. Brunel, Fiona C. Schnitzler, Lisa M. Partel, Hugh S. Paterson, Paul G. Bannon

**Affiliations:** 1 Sydney Imaging Core Research Facility, The University of Sydney, Camperdown, 2006 Australia; 2 The Baird Institute of Applied Heart and Lung Surgical Research, Sydney, Australia; 3 Sydney Medical School, University of Sydney, Camperdown, Australia; 4 University of New South Wales, Randwick, Sydney, Australia

**Keywords:** Cardiac surgical procedures. Temporary left ventricular assist device, Epicardial echocardiography, Ovine model

## Abstract

The Impella CP is a percutaneously inserted temporary left ventricular assist device used in clinical practice and in translational research into cardiogenic shock, perioperative cardiac surgery, acute cardiac failure and mechanical circulatory support. Fluoroscopic guidance is usually used for insertion of an Impella, thus limiting insertion to within catheterization laboratories. Transthoracic, transoesophageal and intracardiac echocardiography have been reported to guide Impella CP implantation with identified specific limitations stemming from the surgical, anatomical and equipment factors. We conducted translational prospective descriptive feasibility investigation as a part of two other hemodynamic Impella studies. It showed the successful application of epicardial echocardiographic scanning for implantation of Impella CP devices in ovine models, from which details of the technique and identified pitfalls are described with practical solutions for future investigators and clinicians. Many described findings are relevant to any other echocardiographic techniques when adequate imaging of the Impella and relevant anatomical structures is achievable.

**Graphical Abstract:**

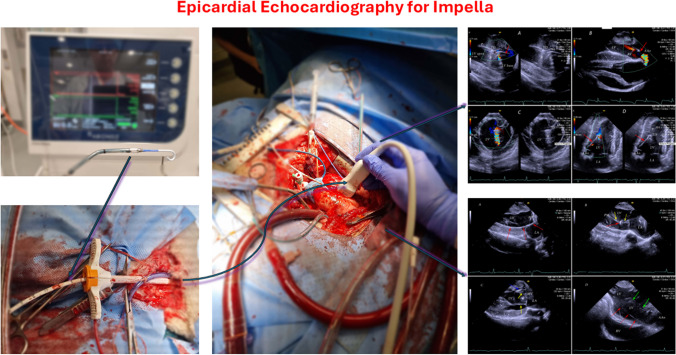

## Introduction

The Impella CP is a left ventricular assist device based on a microaxial pump and is licensed for use for several hours during high-risk percutaneous coronary interventions and up to four days in patients with cardiogenic shock. Successful clinical off-label use for several weeks has been previously reported [1]. Other models of Impella (Impella 5.0 and 5.5) are licensed for left ventricular support up to 14 days and are being progressively used when there is requirement for higher pump flows and an anticipated extended period for circulatory support. The purpose of the Impella CP is to improve cardiac output sufficiently to prevent secondary organ failure and to unload a failing left ventricle (LV). Impella devices are used clinically for temporary mechanical circulatory support during high-risk percutaneous coronary interventions, particularly for the left main coronary artery intravascular interventions, for myocardial infarction associated with severe cardiogenic shock due to the left ventricular failure, in acute viral myocarditis or pharmacologically-induced cardiotoxicity, during off-pump coronary bypass surgery, for post-cardiotomy shock, for supporting the heart in difficult weaning of patients from cardiopulmonary bypass after cardiac surgery, and when there are clinical requirements for left ventricular venting during peripheral veno-arterial extracorporeal oxygenation [2–6]. It is also used in large animal translational research models of mechanical circulatory support [7–9].

An Impella CP is usually inserted in humans percutaneously via femoral or axillary arteries using a modified Seldinger technique with a pigtail guidewire negotiated across the aortic valve into the left ventricle. Large animal models use femoral or carotid artery access. A diagnostic catheter is negotiated over the guidewire into the LV over the guidewire, which is then exchanged for the straight-tipped Impella guidewire. An Impella is then implanted over that guidewire so that the inflow of the device is positioned approximately 3.5 cm below the aortic valve within LV, and the outflow, together with the 14Fr pump motor, above the aortic valve. The 9Fr reinforced Impella catheter traverses the aortic valve, providing continuous flow of blood from the LV into the systemic circulation with a titratable flow rate commensurate with the native cardiac output, up to 3.7 l/min.

Dynamic radiography is the most frequent imaging used to guide the Impella implantation when it is performed in a cardiac catheterisation laboratory or hybrid operating theatre [10]. However, fluoroscopic guidance may not be immediately available in cardiac operating theatres or intensive care units. Fluoroscopy is associated with radiation exposure to the patient and the staff. Echocardiography is usually performed to confirm the device position after fluoroscopically guided placement. Correct positioning of the Impella is critical for optimization of flow and avoidance of complications. Of particular importance is the position of the inflow which may inadvertently impinge on the anterior mitral leaflet, mitral subvalvular apparatus, left ventricular wall or left ventricular outflow tract wall, or migrate into the aortic root. Transthoracic (TTE), transesophageal (TEE) and intracardiac (ICE) echocardiography techniques have been reported to provide adequate imaging for successful and uncomplicated Impella CP placement [11–13]. However, transthoracic and transesophageal echocardiography approaches frequently do not offer adequate views sufficient for safe navigation in ovine and porcine models. Transthoracic echocardiography is impractical in intraoperative cardiac surgery. Although widely utilized during cardiac surgery, in rare circumstances TEE may be inapplicable in the presence of co-existing pathologies, such as oesophageal strictures or recent oral or oesophageal surgery, or severely limited mouth opening, limiting probe insertion in humans. The immediately available epicardial probe can also provide additional views whenever TEE images are suboptimal. This is the case in large animal models where TEE views are often very limited and insufficient for the detailed assessment required for careful implantation of the Impella during cardiac surgery. Reports of intracardiac echocardiography remain scarce due to the lack of equipment and expertise in both clinical and translational research settings [13]. It is also the most invasive of all echocardiographic modalities and requires insertion of a large-bore venous access sheath to allow intracardiac placement of the echocardiographic probe.

Epicardial ultrasound scanning of the aorta is widely used in cardiac surgery prior to cannulation for cardiopulmonary bypass [14] and therefore is readily available in most cardiac surgery institutions, as well as in translational cardiac surgery laboratories. It is also used intraoperatively in coronary arterial surgery, congenital heart disease surgery and for placement of durable ventricular assist devices [15–19]. Detailed recommendations for the human epicardial echocardiographic scanning have been published [20]. The use of epicardial scanning in a large animal model has been previously reported [21].

We hypothesised that it is feasible to guide the implantation of the Impella CP using epicardial echocardiography without the need for fluoroscopy or other echocardiographic techniques.

## Methods

This investigation is a report of recorded experiences during prospective, single-centre translational studies of physiological impacts of the Impella CP, and of extracorporeal circulatory support with veno-arterial extracorporeal membrane oxygenation (VA ECMO), and of the combination of both devices in adult sheep. The parallel studies were approved by the University of Sydney (Australia) Animal Research Ethics Committee (2017/1244, 2019/1650 and 2022/2178) and conducted at the Charles Perkins Centre for Research, The University of Sydney (Sydney, Australia). All studies were performed in accordance with the Australian code for the care and use of laboratory animals.

### Animals

Adult merino ewes received standard acclimatization and premedication. The sheep were anaesthetised and mechanically ventilated with invasive monitoring of arterial pressure, central venous pressure, and cardiac output. Animals received fluid resuscitation and vasopressor support to maintain adequate perfusion pressure. Animals were in the right lateral recumbent position and underwent a left thoracotomy, pericardiotomy and exposure of the heart prior to the Impella implantations, as a part of a parallel study.

### Impella CP

A specialist veterinarian surgeon performed the implantation of the Impella CP as per manufacturer recommendations, using a cut-down approach to the left common carotid artery to provide vascular access. Briefly, a 14Fr 13 cm peel-away introducer was inserted into the left common carotid artery. A stiff 0.035-inch pig-tail guidewire was introduced into ascending aorta and then into the LV through the aortic valve. A 6Fr J-tipped diagnostic catheter was introduced over the wire and advanced into the LV. The 0.035-inch guidewire was removed and replaced with softer 0.018-inch placement guidewire. The diagnostic catheter was removed. The guidewire was loaded in the Impella CP catheter which was then advanced via the carotid sheath into the aorta. The Impella was negotiated over the guide wire through the aortic valve into the LV cavity. The guidewire was removed and the Impella position adjusted by visualizing the tear-drop portion of the catheter in the mid-cavity. The flow was started at the power settings of 2–3 on the Impella controller (corresponding to the average of 1–1.5 l/min pump-generated flow). Once the adequate position was confirmed, the flow was gradually increased to a maximum power setting on the controller (corresponding to 3–3.5 L/minute pump-generated flow).

A pressure–volume conductance catheter (Millar Inc, Houston, TX) was inserted into left ventricle via the LV apex and a pressure catheter (Millar Inc, Houston, TX) inserted into the left atrial cavity via left atrial appendage.

The Impella pump was stopped at the console and then removed completely from the sheep following completion of the study. Animals were then prepared for the immediately subsequent parallel terminal study and euthanised on completion and in accordance with that study protocol.

### Echocardiography

A physician qualified in echocardiography (Advanced Echocardiography training, Level 3) [17] performed all echocardiographic examinations (SC2000, Siemens Healthcare GmbH, Erlangen, Germany) using a cardiac transthoracic two-dimensional phased array TTE probe operating in harmonic frequencies 5–8 MHz and a frame rate of 45–60 f/sec. Two-dimensional greyscale and Color-Doppler imaging modalities were used. Full baseline cardiac assessment was performed via available epicardial views in all cases before initiating implantation of the Impella CP. The baseline assessment included Color Doppler interrogation of the aortic and mitral valves to determine potential pre-existent valvular incompetence and to avoid mistaken iatrogenic attribution of existing pathology following implantation of the Impella, particularly in relation to the aortic valve.

### Implantation sequence

- 1. The first step in implantation process was to advance the 0.035″ guidewire via the valved 14Fr carotid vascular sheath retrograde into the ascending aorta, across the aortic valve and into LV cavity. The footprint of the ultrasound probe was positioned over LV outflow tract with simultaneous cephalad tilting.
- 2. Once the 0.035″ wire crossed the aortic valve, the imaging sector was relocated to the mid-LV with dynamic scanning extending to the apex in intermittently manually adjusted two perpendicular planes. Once the pigtail was visualised at the LV apex, no further advancements of the guidewire were made.
- 3. A 6Fr J-tipped diagnostic catheter was railroaded over the 0.035″ guidewire until its tip reached wire’s tip in the LV apex. The wire was then removed.
- 4. A thin, soft-tipped 0.018-inch guidewire was advanced through the diagnostic catheter. The diagnostic catheter was then removed. The Impella CP was advanced over the thin guidewire under continuous visualization by epicardial ultrasonography. Grey-scale imaging and Color Doppler modalities were used to position the Impella.
- 5. Once the Impella pump was switched on, the Color Doppler imaging in long- and short-axis 2D planes was activated to visualize the blood inlet location and to confirm the Impella position and its relationship with the surrounding cardiac structures.
- 9. The aortic valve was imaged by cephalad and lateral sliding of the footprint of the ultrasound probe, with the imaging plane positioned in-line with the longitudinal ventricular axis and with imaging of the Impella catheter traversing aortic valve. Color Doppler modality was activated to identify the blood outlet of the Impella.
- 10. Manual displacement of the LV apex from the pericardial cradle was performed in several cases to gain apical echocardiographic views in greyscale and Color Doppler ultrasound modalities to better confirm adequate separation of the Impella blood inflow from the mitral apparatus.

### Statistical analysis

This is a descriptive feasibility study for which statistical analyses were not applicable.

## Results

An Impella CP was successfully implanted under epicardial echocardiographic guidance in all fourteen sheep, using two-dimensional greyscale and Color-Doppler modalities. The high-frequency probe was excellent for visualisation of vascular and cardiac walls, valves, subvalvular mitral apparatus including primary and secondary chordae, as well as most elements of the Impella insertion kit. Multiple nuances of epicardial scanning guidance were identified and ratified.

### Pearls and pitfalls

**Fig. 1:**
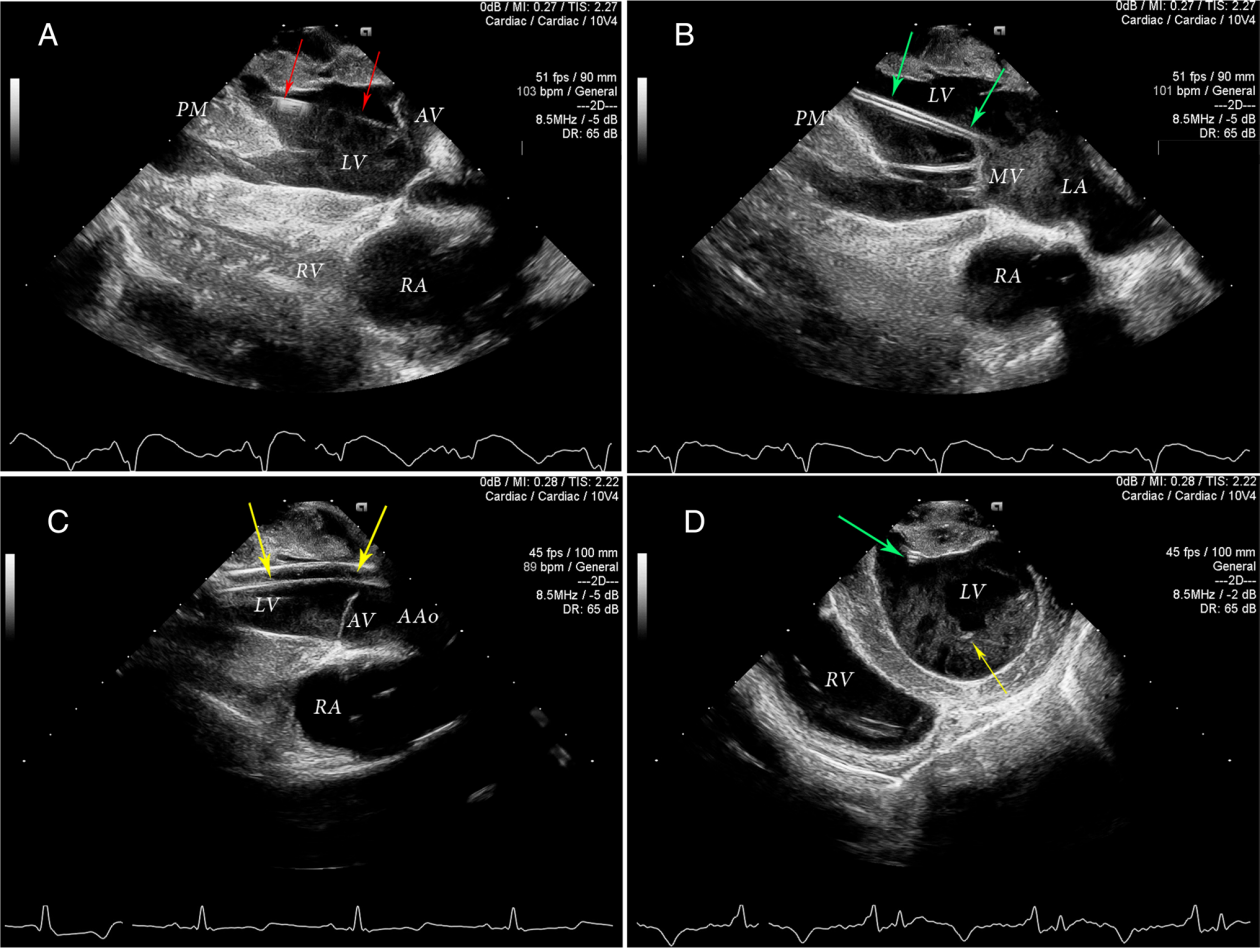
Sequential two-dimensional greyscale epicardial echocardiographic image acquisition in correctly placed elements of Impella CP implantation kit. LV – left ventricle, RV – right ventricle, LA – left atrium, RA – right atrium, AV – aortic valve, MV – mitral valve, Aao – ascending aorta, PM – papillary muscle. **A** Adequate position of the 0.035-inch guidewire (red arrows) across the aortic valve and along LV long axis between PMs, with the tip in LV apex. **B** Adequate position of the diagnostic catheter (green arrows) with longitudinal image projecting from the level of the anterior mitral leaflet towards LV apex. **C** Correct position of the Impella CP (yellow arrows) across the aortic valve and along LV axis. The base and mid- LV cavity is well visualized. **D** Short-axis confirmation view of the Impella CP catheter position (green arrow) adjacent to the base of the anterolateral PM, and the 0.018-inch guidewire (yellow arrow) in the mid-left ventricle

**Fig. 2:**
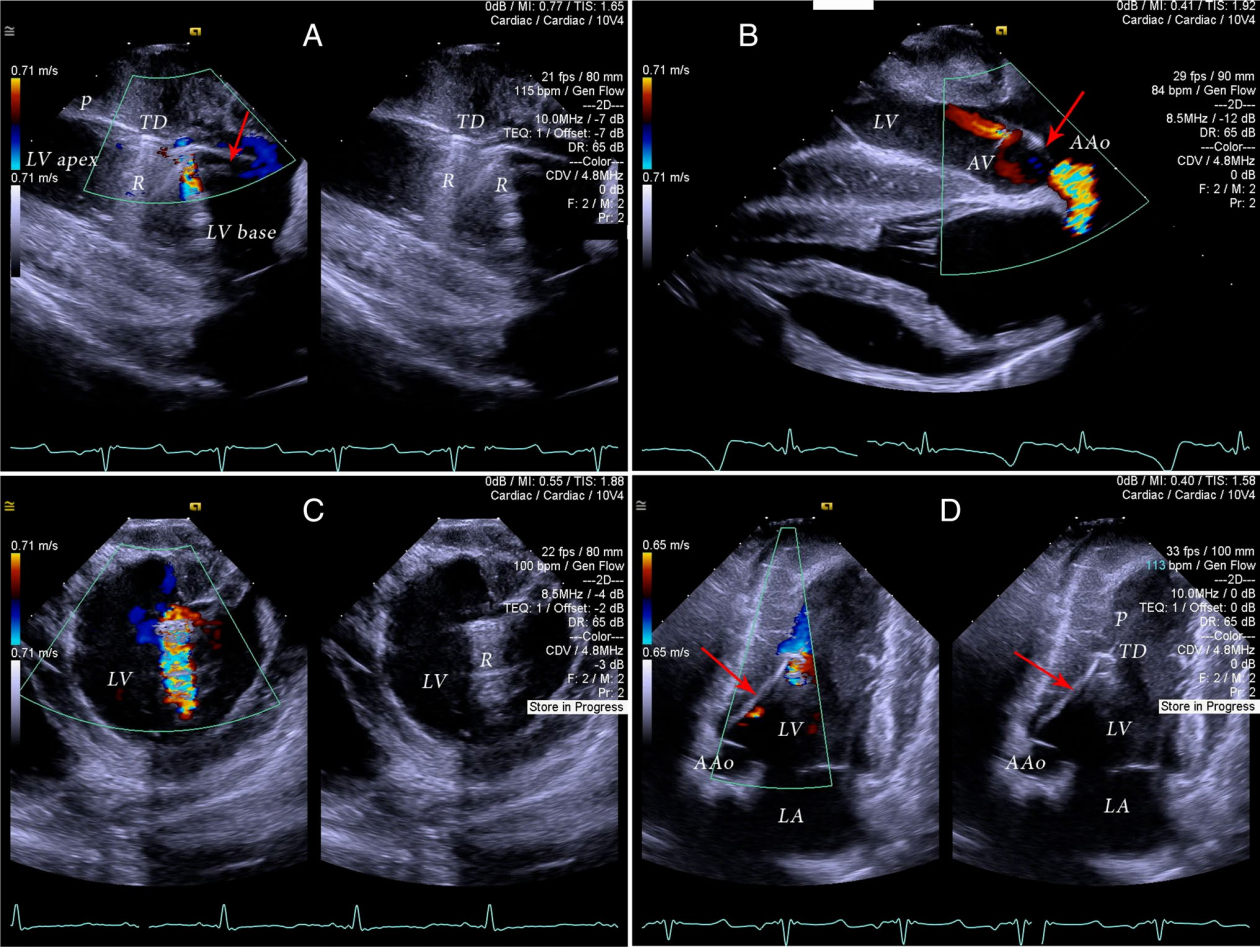
Two-dimensional grey-scale and color-Doppler imaging to confirm correct positioning of the Impella CP. LV – left ventricle, LA – left atrium, AAo – ascending aorta, TD – tea-drop part of the Impella, p- pigtail part of the Impella, R – reverberation artifact. Red arrow points to the Impella catheter traversing towards left ventricular outflow tract and aortic valve. **A** Longitudinal (in-plane) view of the correctly positioned Impella with Color Doppler inflow visualized within mid-LV. Extensive reverberation artifacts originate from the metal tea-drop and from the base of blood inlet area. **B** Longitudinal view of the correctly positioned Impella at the level of the aortic valve and aortic root. The view is an equivalent of inverted 120 TEE view. Color Doppler visualized blood outflow from Impella pump into the proximal ascending aorta. Mild aortic incompetence is visible due to the valvular leaflets coaptation restriction by Impella catheter. **C** Short axis left ventricular view at the level close to the LV base. Color Doppler demonstrated appropriate blood inflow site position of the Impella away from the mitral leaflets and subvalvular apparatus. **D** Apical view, an equivalent of transthoracic 3-chamber apical echocardiographic view. Color Doppler demonstrated adequate Impella blood inflow in mid-left ventricular cavity

**Fig. 3:**
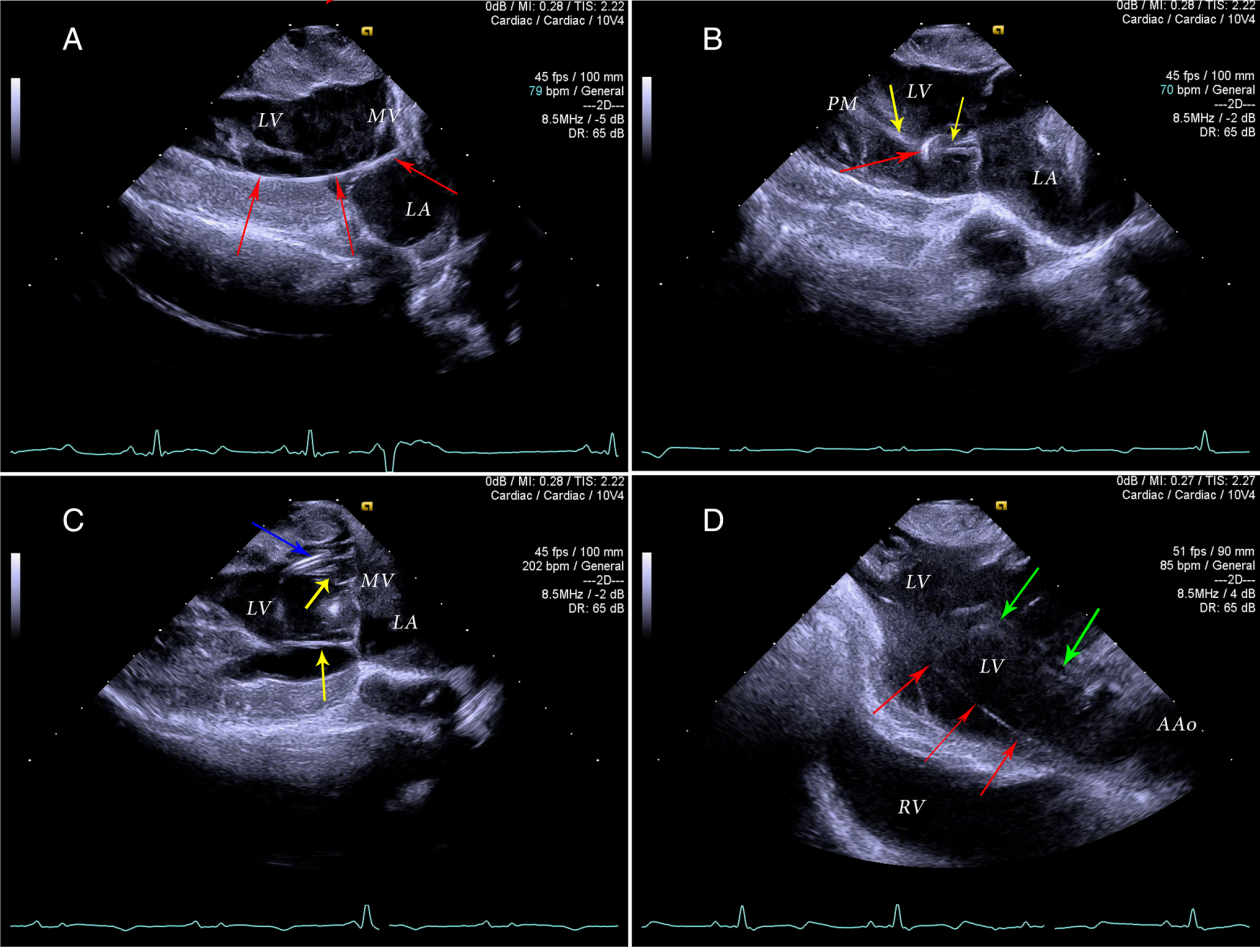
Echocardiographic two-dimensional epicardial imaging of malpositioned insetion kit elements of the Impella. LV – left ventricle, LA – left atrium, RV – right ventricle, MV – mitral valve, PM – papillary muscle. **A** Longitudinal view of the left ventricle and left atrium with stiff 0.035″ J-tip guidewire looping at the LV apex and propagating retrograde into the LA via MV (red arrows). Based on the findings, the wire was withdrawn until de-looped prior to the “railroad” insertion of the diagnostic catheter. **B** Longitudinal view of the LV and LA similar to the parasternal long-axis view obtain by transthoracic echocardiography. The diagnostic catheter has been introduced into position within LV cavity (yellow arrows) over the stiff 0.035″ guidewire, which was excessively pushed into LV during manipulations and looped around diagnostic catheter (red arrow). Partial withdrawal of the diagnostic catheter was required to allow for the J-wire to be carefully removed, avoiding intraventricular knotting. **C** Longitudinal view of the LV and LA following insertion of the thin soft 0.018″ wire via the diagnostic catheter (blue arrow). The diagnostic catheter is well-positioned, while the thin guidewire is excessively inserted, resulting in multiple loops within LV cavity and around mitral subvalvular apparatus (yellow arrows). Careful assessment of the thin guidewire placement is required due to the similar echocardiographic appearance of the device and of the mitral valve chordae, which may lead to the misidentification of parallel structures. **D** Modified longitudinal view of the apical and mid-portions of the LV following placement of the Impella over the soft 0.018″ guidewire. The position of the Impella is adequate (green arrows), while the guidewire is seen looping at the LV apex and propagating towards the ascending aorta (red arrows). Careful withdrawal of the guidewire is required to avoid kinking of the wire at the entry point of the Impella, which may prevent further wire evacuation and would warrant the removal of entire device

- 1(i). Repeated application of ultrasound gel was essential for artifact reduction and image quality. Our terminal studies did not require sterility of the ultrasound probe. It would otherwise be obligatory to use hi-level disinfection of the ultrasound probes and use of sterile plastic sleeves with a sterile coupling ultrasound gel between the sleeve and the heart for intraoperative applications.
- 2(ii). Hyperdynamic states during the periods of hypovolemia and/or inotrope administration induced significant multidirectional rapid bouncing ventricular motion, which made it difficult to keep the probe in sufficiently close contact with myocardial wall throughout the cardiac cycle. Scanning via large blobs of ultrasound gel while stabilising the probe’s footprint 5 mm away from the epicardial surface helped to avoid the LV wall “kicking” the probe and thus optimised the imaging.
- 3(iii). Perma-marking was successfully used on the epicardial surface to tag specific scanning window-points to ensure consistency in repeat examination, especially for quantitative measurements.
- 1(i).The initial scanning point should be adjusted by sliding and tilting the probe over the lateral LV wall to achieve a long-axis LV view. The sector was centred over the base of LV, so that it would capture the LV outflow tract, aortic valve, part of the aortic root, mid-LV cavity, and most of the anterolateral papillary muscle. The right atrium, interventricular septum, and a part of the right ventricle were visualised deeper within the imaging sector (Fig. 1A). Minor translational movements of the probe laterally allowed better viewing of the aortic valve, while medial translational repositioning of the probe better viewing of the mitral valve (Fig. 1B). Moving the probe cephalad opened the aortic root in all cases, and at times allowed visualization of the proximal ascending aorta (Fig. 1C). It was not necessary to visualize the distal ascending aorta and the aortic arch in most cases. A 90° rotation of the probe offered a short-axis view of the LV, a good view of the interventricular septum, and a transverse view of the right ventricle (Fig. 1D). Translational repositioning of the probe along the long LV axis offered parallel multi-slice short-axis views of the LV from the base to the apex.
- 2(ii).Positioning the footprint of the ultrasound probe over the LV outflow tract with simultaneous cephalad tilting was used to bring the imaging sector to the aortic root, allowing excellent visualisation of the guidewire approaching the aortic valve. Occasional difficulty with the first pass of the guidewire across the aortic valve was immediately apparent when the guidewire would not bounce through the valve, but instead would reverse in one of the Valsalva sinuses into the tubular ascending aorta. Re-advancement of the guidewire achieved uneventful AV crossing by the guidewire.
- 3(iii).Placement of the probe footprint at the mid-LV provided excellent views of the guidewire and diagnostic catheter approaching the LV apex. The metallic guidewire was displayed as a bright line with typical reverberation artefact along its length when imaged in-plane (Fig. 1A). The plastic diagnostic catheter was displayed as a hyperechoic double-walled structure, once the guidewire was removed (Fig. 1B).
- 4(iv).Direct visualization of the heart and great vessels provided immediate and uninterrupted points of scanning access via the lateral and anterior LV walls, the antero-lateral left atrial wall, and the aortic root. As a result, a slightly modified equivalent of the left parasternal long-axis and the short-axis mid-papillary and base views were immediately available in all cases. Apical short-axis views were also available but required translational motion of the probe to obtain a true short-axis plane perpendicular to the LV longitudinal axis. The aortic root window was easily accessible in all cases but also required translational movement of the probe under visual guidance over external anatomical landmarks Fig. 2.
- 5(v).Epicardial scanning via the lateral left atrial wall allowed excellent visualisation of the mitral valve, left atrial cavity and a Millar pressure catheter that was surgically implanted in all cases via the base of the left atrial appendage.
- 1(i).Guidewires, catheters, and the Impella were in intimate contact with multiple cardiovascular structures. The potential for inadvertent malpositioning and consequent physical damage to these structures was ever present. Careful multiplane echocardiographic scanning of all elements of the Impella insertion kit at each stage of the implantation is essential for an uneventful procedure. It may require extra time but is the key to avoid iatrogenic complications.
- 2(ii).The 0.035″ pig-tailed guidewire can, despite its stiffness, easily coil within the LV cavity (Fig. 3A). Careful scanning of the guidewire from the entry point via aortic valve into the LV until its tip is clearly identified and repositioned into the LV apex should be done in all cases prior to the introduction of the diagnostic catheter.
- 3(iii).Insertion of the diagnostic catheter can be accompanied by simultaneous inadvertent over-insertion of the stiff guidewire during manipulations (Fig. 3B). The guidewire could then loop around the LV cavity, around the diagnostic catheter and onto itself, creating conditions for forming knots and making its consequent withdrawal difficult or even impossible without vascular injury.
- 4(iv).The soft 0.018″ metal guidewire was not well appreciated in in-line views due to the limited resolution and difficult alignment of its ever-curving directions. In fact, it could be better located within the LV cavity in transverse views (Fig. 1D). The 0.018″ guidewire can appear indistinguishable from the mitral chordae due to the similar thickness and ultrasonographic characteristics. When excessively inserted into the LV cavity (Fig. 3C) and looped around the MV subvalvular apparatus, it created the potential for damage to the chordae and consequent mitral incompetence, especially during attempted withdrawal if knotted on itself. Recognition of this problem is difficult and requires utmost attention from the sonographer.
- 1(i).Excellent imaging of the Impella device and surrounding cardiovascular structures was achieved from the aortic root to the LV apex. The reinforced and slightly curved Impella catheter appeared as a double-walled structure crossing the aortic valve and terminating in mid-LV cavity (Fig. 1C). The position of the Impella catheter and its relationship with the thin guidewire was confirmed on short-axis views by rotating the probe 90 degrees and sliding it caudally (Fig. 1D).
- 2(ii).Both grey-scale imaging and a Color Doppler modality are required to confirm correct positioning of the Impella within the LV cavity. B-mode imaging provided easy identification of the end of the Impella catheter, termed the “teardrop” which is traditionally responsible for an intense echocardiographic reverberation artifact (Fig. 2A). The scanning plane was in-line with the Impella catheter, along the LV longitudinal axis. Color Doppler helped to identify and confirm the correct positioning of the blood inlet, to ensure separation from the mitral chordae with sufficient distance from the mitral leaflets (Fig. 2C).
- 3(iii).Cephalad and lateral sliding of the footprint of the ultrasound probe with the imaging plane positioned in-line with the longitudinal ventricular axis offered good imaging of the ascending aorta and the Impella outlet. Two Doppler jets directed on contralateral sides were seen in most cases (Fig. 2B). The larger and more intense jet directed away from the transducer (along the physiological systolic blood flow direction in the ascending aorta), was found in all cases, and identified throughout the cardiac cycle. It represented the blood outlet from the Impella. Minor repositioning of the outlet orifice was usually required to ensure that the origin of the turbulent outflow jet was positioned within the aortic root. Reduction of the color gain was often needed to minimise color “bleeding” over the surrounding tissues and anatomical structures. A small color Doppler jet, directed towards the transducer was seen in most cases throughout diastole. It represented a degree of aortic incompetence induced by the reinforced Impella catheter positioned across the aortic valve which led to the restriction of complete coaptation of the aortic valve leaflets during diastole, particularly when the intra-aortic pressure was continuously increased by the Impella flow. This jet was located adjacent to the catheter and was consistent with mild aortic regurgitation, except in one with moderate AR (Fig. 2B).
- 5. Apical views could not be obtained without significant foreshortening of the LV due to the position of the apex within remaining pericardium, deeper inside the chest. Manual displacement of the LV from the pericardial cradle was needed to gain apical LV views. From this position, B-mode allowed visualisation of the Impella catheter, metal tear-drop with characteristic reverberation artifacts, and the plastic pigtail part of the Impella device within LV apex. A color-Doppler jet immediately proximal to the tear-drop confirmed positioning of the blood inlet orifice. Physical manipulations of the LV caused temporary haemodynamic disturbance. Therefore, such manipulations should be reserved for cases where uncertainty of Impella positioning remains after alternative scanning windows were used and have proved insufficient. (Fig. 2D).
- 6. At all times, the nature of the two-dimensional imaging used during our epicardial scanning was such that the scanning plane could only capture a part of the three-dimensional cardiac structures and the insertion parts of the Impella kit at any given time. Other parts of the Impella and anatomical landmarks which lie outside of the scanning plane, or those imaged exclusively in the transverse plane, could be missed, misinterpreted or unrecognised. Thus, every element of the Impella insertion kit should be always interrogated in detail and guidewires followed along their length until the tip is clearly identified, to avoid serious malposition.

## Conclusions

Epicardial echocardiography is a feasible and readily available intraoperative option to guide implantation of the Impella CP in ovine models. Understanding the advantages and shortcomings of each available guiding imaging technique, including epicardial scanning, is paramount to avoid serious iatrogenic complications and to improve clinical safety. This translational study is particularly clinically relevant for intraoperative patients who require immediate implantation of the Impella, when other imaging modalities are not readily available or do not offer adequate views for safe guidance of the procedure.

## Data Availability

Study data can be provided on reasonable request.

## Abbreviations

AV: Aortic Valve
LA: Left Atrium
LV: Left Ventricle
LVAD: Left Vetricular Assist Device
LVOT: Left Ventricular Outflow Tract
MV: Mitral Valve
RA: Right Atrium
RV: Right Ventricle
TEE: Transesophageal Echocardiography
TTE: Transthoracic Echocardiography
VA ECMO: Veno-Arterial Extracorporeal Membrane Oxygenation
